# Economic limit calculate method of blocked layer released by water flood wells

**DOI:** 10.1371/journal.pone.0319186

**Published:** 2025-02-24

**Authors:** Fulin Wang

**Affiliations:** E&D Research Institute of Daqing Oilfield Company Ltd, Daqing, China; Universiti Teknologi Petronas: Universiti Teknologi PETRONAS, MALAYSIA

## Abstract

Daqing Oilfield is in the forefront, because of polymer flooding well pattern was used to next production layer, so, first development layer was sealed after polymer well was used to next layer, and whose storage reserves is very huge. To 2021, OOIP of the first set of reserves of type II reservoirs has been sealed up to 9603 × 10^4^ t. In order to release the reserves and reduce the influence on oil yield, water drive well pattern was used to product the sealed reserves through additional perforating, but the economic boundary standard to release the sealed layer using water drive well pattern is not determined, so, based on the principle of break-even and the seepage flow theory, this paper establishes the formula for calculating the economic limits of the various unsealing indexes by water flood well, which can effectively solve the problem of communicating and understanding the economic and engineering parameters of the released well, quantificational the limit of layer selection standard of well for releasing. The result showed the cumulative and daily oil production limit is gradually decreases with oil prices increasing, and vertically, the limit is increases with the input-output ratio (ROI) increases. FWHP of oil well b1-40-527 is 0.4 Mpa, well spacing is 300 m, oil well radius is 0.1 m, viscosity is 8.8 mPa. s, production time of 300 d, density of 0.86 g/cm^3^, when ROI is 1:2, the lower limit of the cumulative oil raised production is 206.9 t and daily increasing oil floor is 0.69 t/d, the minimum released effective thickness is 8.4 m as oil price is 60 $/BBL, meanwhile, the lower limit of the cumulative oil raised production is 421.4 t and daily increasing oil floor is 1.4 t/d when oil price is 40 $/BBL. After increasing the thickness of released layer according the limit, it is verified by actual data, that the increasing oil production of daily and accumulated reached to the calculation minimum limit after thickness increased by re-unsealing. The fluid production daily of the well b1-40-527 was 41.41 t/d and oil production was 1.53 t/d, water cut was 96.3% before the well releasing, then, the well was released firstly in July 2021, total effective thickness released is 6.6 m, after the released, the daily oil production was 1.92 t/d, the daily water cut was 96.3%, and oil production daily increased is 0.39 t/d, which is lower than the requirement of 0.79 t with oil price is 60 $/BBL and ROI is 1:2, therefore, the lower permeability layers were added, the total released thickness is to 10.7 m, which meets the requirement of unsealing, and the daily fluid production of the well b1-40-527 was to 67.79 t/d, the daily oil production was 3.2 t/d, the daily water cut was 95.3%, daily oil production increased was by 1.25 t/d, water cut decreased is 1.0%, and the releasing measure is valid still now.

## Introduction

The petroleum resources in China are distributed in the sedimentary environment dominated by continental strata, most of which are sandstone reservoirs, accounting for more than 90%, it is characterized by serious heterogeneity and high oil density. There are mainly 13 oilfields composed of continental sandstone reservoirs, including Daqing, Shengli, Yanchang, Liaohe, Xinjiang and Changqing oilfields [1–4]. These onshore sandstone oilfields were developed using water flooding and chemical flooding respectively with their own well pattern.

At present, Daqing Oilfield is at the forefront of our country’s oil industry because of the coexistence of the two type displacement method of water and polymer flooding [5–9], improved water drive recovery [10–12] and EOR in oil reservoirs [13,14] is relatively mature, but there are still some problems in well pattern coordination of two type drive formation [15–17], because the polymer flooding well pattern develops production layer independently, the water flooding well pattern needs to block polymer flooding’s layer, the polymer flooding well pattern need to product the neighbor layer when its water cut is higher than 98% [18], which cause the old reserve be sealed [19,20]. Now, reserves of Daqing oilfield’s sealed are the largest and earliest in the country, even in the whole world. By the end of the 2021, 96.03 million tons of second-class reserves had been sealed, at the end of the 14th five-year plan period, storage capacity will reach to 100 million tons. During the 15th five-year and future, Daqing oilfield reserves will store up to one billion tons, and all of the current polymer flood formations will storage due to the utilization of its development wells. The technical and economic limits of the upper and lower return of the second type oil layers are discussed in detail, the problem that the thickness of water drive well pattern is reduced because it block the polymer well production layer, and the production of plugging layer is stopped, but the paper has not given a solution [18], in addition to polymer flooding, measures such as fracturing have an impact on oil development, water-based fracturing fluid has recently garnered increasing attention as an alternative oilfield working fluid for propagating reservoir fractures and transporting sand, increasing reservoir pressure exhibited enhanced thickening and sand-carrying capacities [21,22], and drilling, wellbore stability will also influence oilfield, Li’s research showed increasing mud density or hydrate saturation is beneficial to maintaining wellbore stability in hydrate-bearing sediments, when lower both the mud temperature and salinity, shorten the drilling cycle, and reduce stress difference [23,24], meanwhile reservoir parameters and its get method have influence to economics [25–31]. In this paper, the enhanced oil recovery and development laws of different displacement media (ASP, polymer or polymeric surfactants) are different [32], but our article today focuses on the development methods of oil reservoirs after enhanced oil recovery by ASP flooding or polymer etc. Therefore, we will study the relevant parameters of unsealing development using water drive wells, for example, thickness and permeability of unseal layer, or, viscosity and density of crude oil, and integrate utilization of existing well patterns to release the sealed reserves and productivity, which are using and applied in Daqing oilfield, the technology of release the sealed reserves used in Daqing is a leading position in China.

In order to release the potentiality of the sealed reserves and reduce the influence on oil production, the synthesize utilization of the existing well pattern to release the reserves is an effective and benefit method, that is, the sealed post-chemical flooding reserves are effectively developed by water flooding well re-perforate, but, there is no clear thickness limits, the economic effective and validity of oil increment has not be accurately definition either, so the paper carried out this study for better guide the re-perforating layer optimization of release well.

For determine the economic boundary standard of unsealing by water drive well, the economic boundary formula for calculating the unsealing indexes is established through the break-even principle. The advantage of break-even is that it is the simplest method in uncertainty analysis [33], by calculating the balance relationship between quantity, cost, and profit, the lower limit of different influencing factors can be determined by finding the equilibrium point. The disadvantage comes from the assumption conditions established by this method, because the equilibrium point requires the assumption that sales volume equals output volume, when calculating any indicator, other factors are assumed to be constant and known, which is very ideal and not easy to meet in practice, it is easy to obtain the minimum profit oil production using breakeven analysis only, we can assume some constant factor exchange rate is 7.0 of RMB against the US dollar, as we know, ton-to-barrel conversion rate is 7.14, and crude oil commodity rate is 98.37%.

However, it is impossible to describe the impact of the production process effect to oil production, therefore, using a combination of breakeven and seepage flow mechanics can effectively combine economic oil production with reservoir engineering, and guide production effectively, it is indeed a practical analysis method and the theory of seepage flow, we can effectively solve the problem of communication by formula, which understand the economic and engineering parameters of unsealing well, quantify the standard boundary of sealing well and layer selection, which fill up the blank of theory and method about layer boundary after chemical flooding by using water flooding well, it can provide support for the releasing of Daqing oilfield’s sealed reserves and has important guiding significance for the development of multilayer sandstone reservoirs.

## Method establishment

In order to determine the economic boundary standard for unsealing wells belong to water drive well pattern, the calculation formula of economic boundary for unsealing wells is established through the principle of break-even principle and seepage flow method, so, floor level of the thickness and cumulative oil production of unsealing well is determined.

### The economic boundary formula

When the marginal cost is equal to the marginal revenue, and the cost of the product produced is equal to the selling oil price, the calculated limit is yield index limit. Marginal cost includes two parts: one is the measure cost needed to increase crude oil production, and the other is the variable cost of per unit crude oil production.


$$M_{c}=\frac{\Delta C_{m}}{aN_{p}}+C_{v}$$ (1)

Marginal revenue is oil price minus taxes, calculated as:


$$M_{s}=P-T_{r}$$ (2)

According to the break-even principle, when the ratio of the average marginal cost to the oil price deduct tax is equal to the required ROI, the corresponding minimum cumulative incremental oil production is the economic limit of the incremental oil production by unsealing measures.

That is:


$$M_{c}/M_{s}=R$$ (3)

Thus, the limit of oil increment for unsealing measures is:


$$N_{pmin}=\frac{\Delta C_{m}}{(P-T_{r})aR-aC_{v}}$$ (4)

### Yield formula in percolation mechanics

In Darcy’s law:


$$v=\frac{k}{\mu }\cdot \frac{\text{d}p}{\text{d}r}$$ (5)

The well production formula for planar radial flow:


$$Q=A\cdot v=2\pi rh\cdot \frac{k}{\mu }\cdot \frac{\text{d}p}{\text{d}r}$$ (6)

According to the seepage theory, the production of oil wells is based on the above formula, and considering the relative permeability and volume coefficient of the oil phase, it is converted into the following equation:


$$Q_{\text{o}}=\frac{2\pi hkk_{\text{ro}}(p_{\text{i}}-p_{\text{o}})}{\mu B_{\text{o}}\ln\frac{R_{\text{e}}}{r_{\text{w}}}}$$ (7)

The production unit in the above equation is volume unit, multiplied by density ρon the right side, converted to weight units, 86.4 in the formula is the coefficient generated after unit standardization:


$$Q_{\text{o}}=86.4\cdot \frac{2\pi hkk_{\text{ro}}(p_{\text{i}}-p_{\text{o}})}{\mu B_{\text{o}}\ln\frac{R_{\text{e}}}{r_{\text{w}}}}\cdot \rho$$ (8)

The cumulative oil production formula for the effective period of the measures is obtained:


$$N=Q_{\text{o}}\cdot D=86.4\cdot \frac{2\pi hkk_{\text{ro}}(p_{\text{i}}-p_{\text{o}})}{\mu B_{\text{o}}\ln\frac{R_{\text{e}}}{r_{\text{w}}}}\cdot \rho \cdot D$$ (9)

### Unseal the economic boundary formula

From formula (4) and (9), the formula of unsealing index can be used to calculate the economic boundary under different hole-filling parameters.


$$86.4\cdot \frac{2\pi hkk_{\text{ro}}(p_{\text{i}}-p_{\text{o}})}{\mu B_{\text{o}}\ln\frac{R_{\text{e}}}{r_{\text{w}}}}\rho \cdot D=\frac{\Delta C_{\text{m}}}{(P-T_{\text{r}})aR-aC_{\text{v}}}$$ (10)

## Results and discussion

### Lower limit of oil production accumulated and daily increasing

The block B1dd is located in Daqing oilfield’s Sazhong development zone. The second-class oil layers of the block mainly developed SP (Sartu and Putaohua) oil layers, which are sand-mudstone interlayers deposited by River-delta, with severe heterogeneity. From top to bottom, they are the S oil layer and the P oil layer. The two oil reservoir were divided into 5 oil layer groups, 15 sandstone groups, 45 sublayers, and 58 sedimentary units. The block was started development in June 196, and has undergone five adjustments. Currently, the SP oil layers are divided into four series for exploitation, namely, the basic well network, the first well-infill adjustment network, the second well-infill adjustment well network, and the PI group polymer drive well network. In order to verify the use of wells in this block in the future, the parameters used for this theoretical calculation are the development parameters and reservoir parameters of this block, which facilitates the comparison between theory and practice. Perforate costs of a well in block is 20 × 10^4^ yuan, the average water injection pressure of the block is 10 MPa, permeability of the production layer are 300 × **10**^**-3**^ *μm*^*2*^, oil viscosity is 8.8 mPa.s obtained from block B1dd, flowing well head pressure (FWHP) is 0.4 MPa got from well B1-40-527. Using the calculation parameters in Table 1, namely, the tax per ton of oil is 410 yuan, the commodity rate is 98.37%, the variable cost is 312.55 yuan, and the releasing cost is 20 × 10^4^ yuan for calculation.

**Table 1:** Parameters used in model calculations of B1dd block.

Tax (Yuan/t)	Commodity rate (%)	Variable costs (Yuan/t)	Perforate costs (Yuan)	Injection pressure (MPa)	FWHP (MPa)	Oil Viscosity (mPa.s)	Production days (d)	Permeability (10^-3^ μm^2^)	Well Space (m)	Oil Density (g/cm^3^)	Oil phase relative permeability (water cut 98.1%)	Crude oil volume ratio
410	98.37	312.55	200000	10	0.4	8.8	300	300	300	0.86	0.006	1.3

According to the derived calculation formula, substitute the above parameters into formula 4:


$$N_{\text{pmin}}=\frac{200000}{(P-410)\times 0.9837\times R-0.9837\times 312.55}$$ (11)

**Fig 1:**
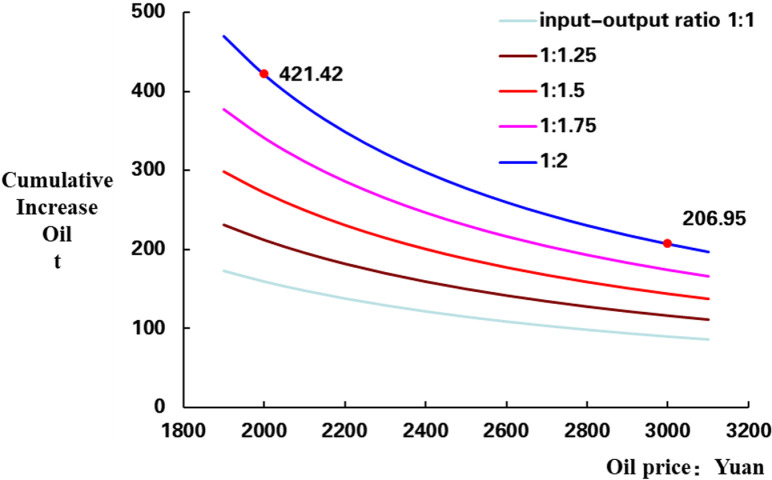
Cumulative increasing oil limit of different oil prices and ROI.

**Fig 2:**
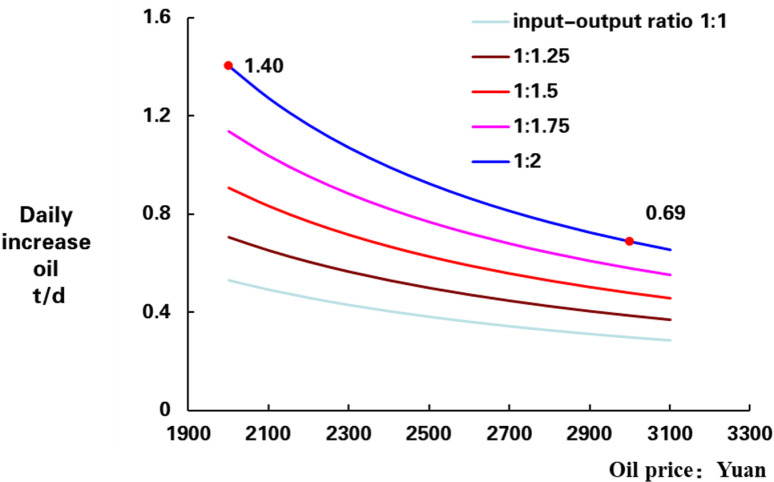
Daily increased oil limit of different prices and ROI.


According to different oil prices *P* and ROI *R*, the corresponding lower limit of cumulative oil production can be obtained. For example, when the oil price is 60 $/BBL, and when converted to RMB at an exchange rate of 7.0, it is about 3000 yuan. 1 ton is 7.14 BBL, when the ROI is 1:2, calculated cumulative oil production limit is 207 t, and then, the curve of cumulative increasing oil with different oil prices and ROI is obtained (Fig 1). When production days are 300 d, we can get daily oil increasing oil curve (Fig 2).

$$N_{\text{pmin}}=\frac{200000}{(3000-410)\times 0.9837\times 0.5-0.9837\times 312.55}=206.9459\text{t}$$ (12)

$$Q=\frac{N_{\text{pmin}}}{300}=\frac{206.9459}{300}=\text{0.6898 t/d}$$ (13)

In the Fig 1, the x-axis is the oil price, the y-axis is the cumulative increase oil production (CIOP), and, there are 5 curves in chart of different ROI, the lowest one is ROI 1:1 and the highest one is 1:2. Horizontally, as oil price increasing, the CIOP limit is gradually decreases, in the case ROI is 1:2, the lower limit of CIOP is 206.9 t when oil price is 60 $/BBL (3000 CNY), and the limit of CIOP is 421.4 t when oil price is 40 $/BBL (2000 CNY), that is bigger than 60 $/BBL. Vertically, the CIOP limit is increases with the ROI increases, as oil price is 40 $/BBL, the lower limit of CIOP is 421.42 t when ROI is 1:2, that is bigger than ROI 1:1 (Fig 1).

In the Fig 2, the x-axis is the oil price, the y-axis is the daily increase oil production (DIOP), and, there are 5 curves in chart of ROI, the lowest one ROI is 1:1 and the highest one ROI is 1:2. Horizontally, as oil price increasing, the DIOP limit is gradually decreases (Fig 1), in the case of ROI is 1:2, the lowest limit of DIOP is 0.69 t/d when oil price is 60 $/BBL, and it is 1.4 t/d, when oil price is 40 $/BBL, that is bigger than 60 $/BBL. Vertically, the DIOP limit is increases with the ROI increases, as oil price is 40 $/BBL, the lower limit of CIOP is 1.4 t/d when ROI is 1:2, that is bigger than ROI 1:1 (Fig 2).

#### 2.4.2. Lower limit of release thickness.

From Table 1, permeability is 300 × 10^-3^ μm^2^ in, injection pressure is 10 MPa of injection well, FWHP of oil well is 0.4 MPa, well spacing is 300 m, oil well radius is 0.1 m, viscosity is 8.8 mPa. s, production time of 300 d, density of 0.86 g/cm^3^ are substituted into formula 10. The lower limit of releasing well thickness is calculated based on the calculation parameters and process in 2.4.1.


$$86.4\times \frac{2\pi h\times 0.3\times 0.006\times (10-0.4)}{8.8\times 1.3\times \ln(\frac{300}{0.1})}\times 0.86\times 300=\frac{200000}{(P-410)\times 0.9837\times R-0.9837\times 312.55}$$ (14)

**Fig 3:**
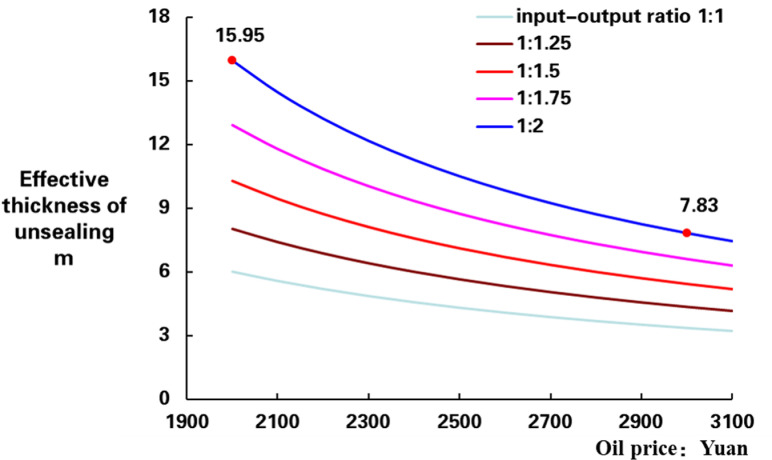
The thickness limit of supplementary with different input and output in oil wells.

According to the equation above, when oil price is 60 $/BBL and the ROI is 1:2 with an exchange rate of 7.0, the cumulative oil production limit is calculated to be 206.9 t. Based on production time of 300 d, the thickness lower limit can be calculated to be 7.8 m. The thickness lower limit chart for different oil prices and ROI can be obtained (Fig 3).

In the Fig 3, the x-axis is the oil price, the y-axis is effective thickness of unsealing (ETU), there are 5 curves in chart of ROI, the lowest one ROI is 1:1 and the highest one ROI is 1:2. Horizontally, the ETU limit is gradually decreases with oil price increasing, in the case of ROI 1:2, the ETU limit is 15.95 m, calculated based on an oil price of 40 $/BBL, and the ETU is 7.83 m with oil price is 60 $/BBL smaller than oil price 40 $/BBL. Vertically, the smaller ROI, the littler ETU limit, as oil price is 40 $/BBL, the lower limit of ETU is 15.95 m with ROI is 1:2, that is bigger than ROI 1:1 (Fig 3).

## Application

### Basic situation

The block B1dd is located in daqing oilfield’s Sazhong development zone. There are four sets of well patterns in the SP formation, the SII1-9 formations were developed in 2019 by upward of polymer flood well of the SII10-SIII10 series, as a result, the reserves of SII10-SIII10 formation are sealed, and the water cut of SII10-SIII10 formation is 97.97% when sealed.

**Fig 4:**
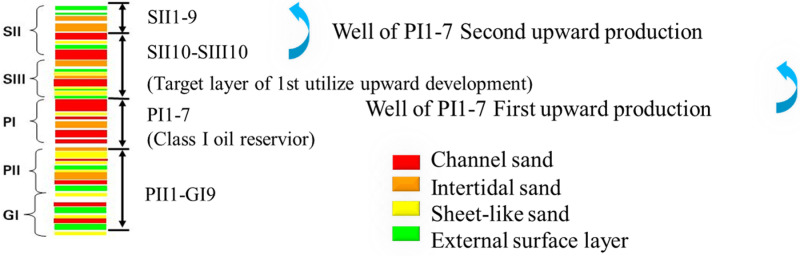
Upward return patterns of same set well pattern in BB1 block.

**Fig 5:**
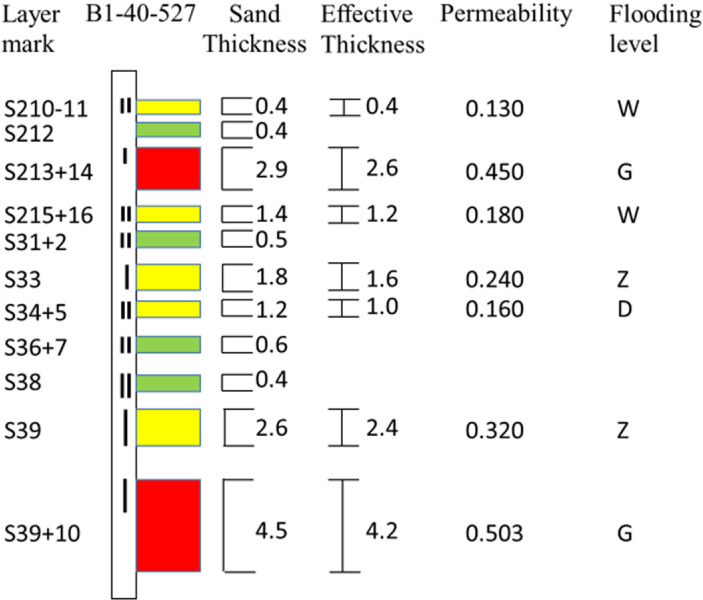
B1-40-527 well log interpretation results and perforation schematic diagram.

Well B1-40-527 is an oil well in the water drive section of the block, and develops the SII and PII layer sections. The SII10-SII10 sections of the well (Fig 4) had been blocked in coordination with the polymer flood well production. The thickness of the sandstone in SII10-SII10 section is 16.7 m, and the effective thickness is 13.4 m, the effective permeability is 283 md, the effective thickness of post water-flooded is 6.8 m, the effective thickness of non-water-flooded is 1.6 m (Fig 5).

### According to the boundary of calculation, the optimum location of reperforating and unsealing is selected

According to the interpretation results of B1-40-527 well log, plugging situation and layer thickness, permeability and water cut of unperforated layer in SII10-SIII10 target zone, combined with logging curve and remaining oil analysis, four layers (S213+14, S33, S39, S39-10) were selected to release (the I perforating part in Fig 1), which total effective thickness is 6.6 m. The fluid production daily was 41.41 t/d and oil production was 1.53 t/d, water cut was 96.3% before the well releasing. Then, the well released the four layers in July 2021, after the released, the daily fluid production was 52.3 t/d, the daily oil production was 1.92 t/d, the daily water cut was 96.3%, oil production daily increased is 0.39 t/d, which is lower than the requirement of 0.79 t with oil price is 60 $/BBL and ROI is 1:2.

So, the lower limit of unsealed thickness is calculated according to Chapter 2.2, the thickness of unsealing should be greater than 8.4 m, and the chosen 6.6 m thickness selected can’t meet the limit requirement. Therefore, the lower permeability layers S210-11, S215+16, S31+2, S34+5 were added (the II perforating part in Fig 5), meantime, the extremely low permeability S36+7, S38 layers were increased, the total released thickness is to 10.7 m, which meets the requirement of unsealing. Then we verify the increment of oil production to determine whether the method is reliable.

### According to oil increasing after releasing thickness added, the method is reliable and feasible

The b1-40-527 well released the blocked layer in May 2022. The thickness was 10.7 m. before released, the fluid production daily was 51.41 t/d and oil production was 1.95 t/d, water cut was 96.3%. After the re-unsealing, the daily fluid production was 67.79 t/d, the daily oil production was 3.2 t/d, the daily water cut was 95.3%, daily oil production increased is by 1.25 t/d, water cut decreased is 1.0%, and is still valid now, cumulative oil production reached to 651.2 t, both reaching the theoretical limit of ROI 1:2 under 60 $/BBL, even exceeding the requirement under 40 $/BBL, it is proved that the re-perforated layer is effective, and the method to determine the economic limit of release reservoir using water drive wells is feasible.

## Conclusions

(1) For the first time, based on the profit and loss balance principle and seepage theory, an economic limit formula for calculating unsealing indexes is established, by using this formula, the economic and engineering parameters of the sealed layer system after chemical flooding are communicated effectively, and the standard boundary of layer selection is quantified.
(2) When the ratio of input to output is 1:2, the minimum release thickness is 7.8 m at oil price is 60 $/BBL. It can be seen that with the increase of oil price, the re-perforate thickness limit decreases, and ratio is smaller, the smaller thickness limit.
(3) According to the unsealing limit of the actual well, after the increase of the thickness, the daily oil and accumulated oil production increasing reach the minimum limit of the theory. This method can effectively guide the thickness limit of the releasing well, it fills up the blank of theory and method about the policy boundary of chemical flooding after unsealing by water drive well.

**Table:** Nomenclature

*M*_*C*_	Measure average marginal cost, yuan/t
△C_m_	Measure cost, including measure operating cost and equipment cost shared during the validity period, yuan
*N*_*p*_	Cumulative oil increment during the effective period of the measures, t
C_v_	Variable cost per ton of oil, yuan/t
a	crude oil commodity rate, %
*M*_S_	Marginal revenue per ton of oil, yuan/t
*P*	Oil Price, Yuan/t
*T*_r_	Tax per ton of oil, Yuan/t
*Q*	Daily oil production, t
*N*	Cumulative oil yield, t
*h*	Seepage thickness, m
*k*	Permeability, 10^-3^ μm^2^
*R*_e_	injection-production well distance, m
*r*_w_	Well radius, m
D	Days of production
*P*_i_	Injection pressure, MPa
*P*_o_	Oil well head flow pressure, MPa
*K*_ro_	Relative permeability of oil phase, *f*
*ρ*	Crude oil density, g/cm^3^。
*B*_O_	Crude oil volume coefficient, *f*。
